# The expression, clinical relevance, and prognostic significance of HJURP in cholangiocarcinoma

**DOI:** 10.3389/fonc.2022.972550

**Published:** 2022-07-28

**Authors:** Yang Yang, Jinyan Yuan, Zhenzhong Liu, Wenwen Cao, Pei Liu

**Affiliations:** ^1^ Department of Hepatobiliary Surgery, The Second Affiliated Hospital of Shandong First Medical University, Tai’an, China; ^2^ Department of Respiratory Medicine, The Second Affiliated Hospital of Shandong First Medical University, Tai’an, China; ^3^ Department of Burn and Plastic Surgery, Qilu Hospital Affiliated to Shandong University, Jinan, China

**Keywords:** HJURP, cholangiocarcinoma, prognosis, biomarker, CCA subsets

## Abstract

**Background:**

Cholangiocarcinoma (CCA) is the malignancy originating from the biliary epithelium, including intrahepatic (iCCA), perihilar (pCCA), and distal (dCCA) CCA. The prognosis of CCA is very poor, and the biomarkers of different CCA subsets should be investigated separately. Holliday junction recognition protein (HJURP) is a key component of the pre-nucleosomal complex, which is responsible for normal mitosis. The ectopic expression of HJURP has been reported in several cancers, but not CCA.

**Materials and methods:**

In our study, we investigated the expression of HJURP in 127 CCA patients which were composed of 32 iCCAs, 71 pCCAs, and 24 dCCAs with immunohistochemistry and divided these patients into subgroups with a low or high expression of HJURP. With chi-square test and univariate and multivariate analyses, we evaluated the clinical relevance and prognostic significance of HJURP in iCCAs, pCCAs, and dCCAs.

**Results:**

HJURP was ectopically upregulated in CCAs compared with the para-tumor tissues based on TCGA and other mRNA-seq databases. A high expression of HJURP was correlated with low overall survival rates of iCCA and pCCA, but not in dCCA. Moreover, HJURP was an independent prognostic biomarker in both iCCA and pCCA. Patients with high HJURP were more likely to suffer CCA-related death after operation.

**Conclusions:**

HJURP was an independent prognostic biomarker in both iCCA and pCCA, but not in dCCA. Our results provide more evidence of the molecular features of different CCA subsets and suggest that patients with high HJURP are more high-risk, which can guide more precision follow-up and treatment of CCA.

## Introduction

Cholangiocarcinoma (CCA) is the malignancy originating from the biliary epithelium and has molecular features of cholangiocyte differentiation (1, 2). CCA is categorized as intrahepatic (iCCA), perihilar (pCCA), and distal (dCCA) CCA according to different anatomical locations, which have distinct epidemiology, oncological behaviors, treatment options, and prognoses (3, 4). The pCCA and dCCA were also defined as extrahepatic CCA (ehCCA), till the 7th AJCC/UICC separated them as distinct CCA subtypes. The outcome of CCA is very poor because the early symptoms of CCA are silent and CCA is not very sensitive to radiotherapy and chemotherapy. The studies of CCA on the field of biomarkers, genetic alterations, drug therapies, and treatment strategies are far away behind other tumors such as hepatocellular carcinoma. Recent advances in high-throughput sequencing have depicted the genetic landscape of CCA and identified different CCA molecular patterns in the genome and transcription levels (5–7). However, the changes at protein levels and posttranscriptional levels of CCA have not been well elucidated.

The centromere is essential for precision and equal segregation, and centromere protein-A (CENP-A) is a unique histone H3 variant responsible for organizing functional centromeres (8, 9). As a molecular chaperone of CENP-A, holliday junction recognition protein (HJURP) mediates CENP-A deposition by forming a pre-nucleosomal complex with the CENP-A/H4 heterodimer *via* its highly conserved Scm3 domain (10–13). The function of HJURP in centromeres is essential to maintaining chromosome precision separation and mitosis (12). As for cancer progression, HJURP was reported to promote progression in several cancer types including hepatocellular carcinoma, prostate cancer, pancreatic cancer, and glioblastoma (14–16). As a diagnostic or prognostic biomarker, HJURP expression is relevant with the prognosis of several cancer types including colon cancer, breast cancer, and hepatocellular carcinoma (17–19). However, the expression, clinical relevance, and prognostic significance of HJURP in CCA are still totally unknown.

In our study, we investigated the expression of HJURP in 127 CCA patients comprising 32 iCCAs, 71 pCCAs, and 24 dCCAs with immunohistochemistry (IHC) and divided these patients into subgroups with a low or high expression of HJURP. With chi-square test and univariate and multivariate analyses, we evaluated the clinical correlation and prognostic significance of HJURP in iCCAs, pCCAs, and dCCAs.

## Materials and methods

### Patients and ethics

Our cohort was composed of 127 consecutive CCA patients who underwent radical surgery and received regular follow-up. The cohort consisted of 67 male patients and 60 female patients. All the specimens were obtained with the prior consent of patients, and the study was approved with the Ethics Committee of Qilu Hospital of Shandong University and the Second Hospital Affiliated to Shandong First Medical University. The tumors were classified and staged according to the 8th edition of the AJCC/UICC TNM classification system.

### TMA and IHC detection

Tissue microarray (TMA) was used for IHC detection. Hematoxylin and eosin (HE) staining was performed to confirm the diagnosis and design the expected area for TMA histological features of all samples. Core biopsies 1.0 mm in diameter were taken from each sample and arranged into TMA slides.

For IHC, TMA slides were first de-paraffined and rehydrated and then incubated in sodium citrate buffer (pH = 6.0), which was boiled by a microwave for optimal antigen retrieval. After that, TMA slides were immersed in hydrogen peroxide for 15 min to minimize the endogenous peroxidase activity. After incubation in 5% bovine serum albumin for 30 min to inhibit the unspecific binding, the TMA was incubated in a primary antibody (Abcam, Cambridge, UK, catalog: ab100800) at 1:50 overnight at 4°C and then in a secondary antibody (Beyotime, Beijing, China) at room temperature for 1 h. DAB was finally applied for the visualization of antigen.

### IHC results evaluation

The tumor area was selected by a senior pathologist, and the IHC results of the tumor area were semi-qualified by the IHC score, which was the product of stained cells and staining intensity. The stained cells were classified as <25%, 25%–50%, 50%–75%, and ≥75%, representing scores 1, 2, 3, and 4, respectively. The staining intensity was stratified as negative, weak, moderate, and strong staining, representing scores 0, 1, 2, and 3, respectively. Therefore, the final IHC score was set from 0 to 12. The cutoff was defined with the receiver operating characteristic (ROC) curve and applied to divide the cohort into high- or low-HJURP subgroup.

### Statistical analysis

Statistical significance was analyzed with software SPSS 22.0. The correlation between HJURP and clinicopathological factors was assessed by the chi-square test. The Kaplan–Meier method was applied to plot the survival curves, and the log-rank test was administrated to generate the statistical significance of different subgroups. The independent prognostic significance of HJURP and other clinicopathological factors was estimated with multivariate analysis by the Cox proportional hazards regression model. *P* value < 0.05 was considered statistically significant.

## Results

### Basic information of the CCA cohort

From 2014 December to 2019 May, a retrospective CCA cohort was established, consisting of 127 CCA patients who underwent radical surgery. This cohort was composed of 32 iCCAs, 71 pCCAs, and 24 dCCAs (**Table 1**). A total of 67 male patients and 60 female patients were enrolled, with the average age of 55.8 years and the average follow-up time of 15.8 months. The 3-year overall survival (OS) rate of this cohort was 16.1%, and the average OS time was 20.7 months.

**Table 1:** Basic information of the CCA cohort.

Clinicopathological parameters		iCCA		pCCA		dCCA	
		n	Percentage	n	Percentage	n	Percentage
Age (years)	<60	20	62.50%	41	57.75%	14	58.33%
	≥60	12	37.50%	30	42.25%	10	41.67%
Gender	Male	16	50.00%	42	59.15%	9	37.50%
	Female	16	50.00%	29	40.85%	15	62.50%
Tumor size (cm)	<3cm	6	18.75%	56	78.87%	17	70.83%
	≥3cm	26	81.25%	15	21.13%	7	29.17%
Histological grade	I	3	9.38%	13	18.31%	1	4.17%
	II	17	53.13%	50	70.42%	16	66.67%
	III	12	37.50%	8	11.27%	7	29.17%
T stage	T1	7	21.88%	10	14.08%	2	8.33%
	T2	18	56.25%	44	61.97%	12	50.00%
	T3	1	3.13%	15	21.13%	8	33.33%
	T4	6	18.75%	2	2.82%	2	8.33%
N stage	N0	19	59.38%	44	61.97%	19	79.17%
	N1	13	40.63%	27	38.03%	5	20.83%
TNM stage	I	7	21.88%	14	19.72%	5	20.83%
	II	10	31.25%	29	40.85%	10	41.67%
	III	8	25.00%	26	36.62%	7	29.17%
	IVA	7	21.88%	2	2.82%	2	8.33%
HJURP	Low	15	46.88%	23	32.39%	7	29.17%
	High	17	53.12%	48	67.61%	17	70.83%

iCCA, intrahepatic cholangiocarcinoma; pCCA, perihilar cholangiocarcinoma; dCCA, distal cholangiocarcinoma; HJURP, holliday junction recognition protein.

### The expression of HJURP in different CCA subtypes

**Figure 1:**
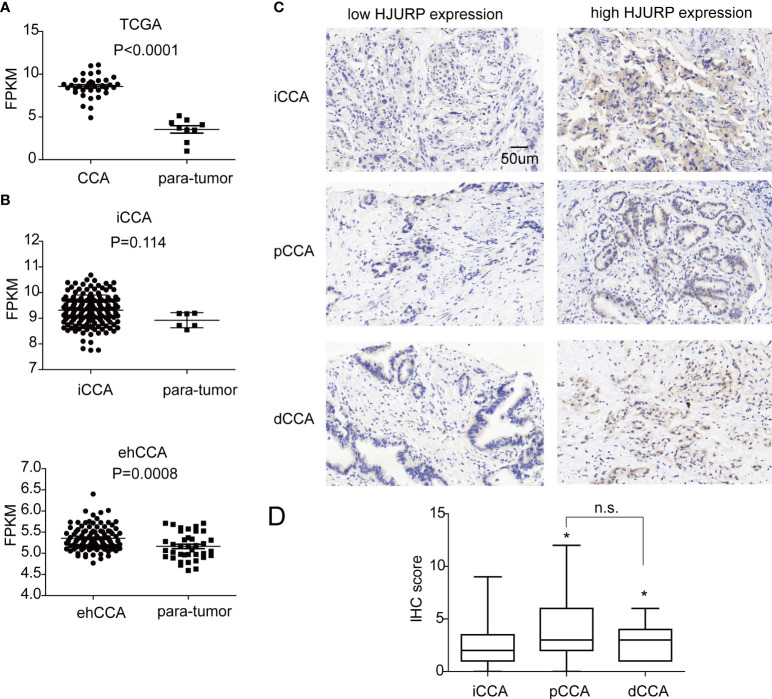
The expression of HJURP in CCA. **(A)** The fragments per kilobase of exon model per million mapped fragments (FPKMs) of HJURP in CCAs and para-tumor tissues in TCGA database. **(B)** The FPKMs of HJURP in iCCAs, ehCCAs, and their para-tumor tissues in the database with GSE33327 and GSE132305. **(C)** HJURP expression in CCAs was detected with IHC, dividing the patients into low and high HJURP expressions. **(D)** The IHC scores of pCCAs and dCCAs were higher than iCCAs. * represents *P* < 0.05, and n.s. represents not significant. In A, B, and D, statistical significance was analyzed by t test.

According to TCGA database and GEPIA, HJURP was upregulated in many types of cancers compared with the para-tumor tissues, including bladder urothelial carcinoma, breast cancer, and colon cancer (20). In CCA, HJURP was also highly expressed than para-tumor tissues (**Figure 1A**). Moreover, we retrieved the mRNA-seq data of iCCA (GSE33327) and ehCCA (GSE132305) from the GSE database (5, 6). Interestingly, HJURP was overexpressed in ehCCA than para-tumor tissues, but this tendency was not statistically significant as to iCCA (*P* = 0.114)(**Figure 1B**).

Furthermore, the expression of HJURP was detected with IHC, and the patients were divided into subgroups with a low or high expression of HJURP. The ratios of low or high expression of HJURP in iCCA, pCCA, and dCCA were 46.88% vs 53.12%, 32.39% vs 67.61%, and 29.17% vs. 70.83%, respectively (**Table 1**). It is interesting to note that most cases had an HJURP expression in the cytoplasm instead of the nucleus, which may be in conflict with its function as a CENP-A chaperone (**Figure 1C**). Moreover, the expression of HJURP was semi-qualified by IHC scores. Intriguingly, the IHC scores of pCCA and dCCA were significantly higher than the HJURP scores of iCCA (**Figure 1D**), indicating that iCCA, pCCA, and dCCA had different oncological characters.

### Clinical relevance of HJURP in CCA

Several clinicopathological factors including the gender and age of patients, tumor size, histological grade, tumor infiltration (T stage), lymphatic invasion (N stage), and TNM stage were enrolled to investigate the potential HJURP-associated factors in CCA (**Table 2**). As a result, a high HJURP was significantly associated with the advanced T stage of iCCA, suggesting that HJURP may participate in iCCA proliferation and infiltration. Moreover, the associations of HRJUP with patients’ gender (*P* = 0.077) and histological grade (*P* = 0.059) in iCCA, tumor size (*P* = 0.076), and N stage (*P* = 0.089) in pCCA as well as tumor size in dCCA (P = 0.058) approached to be statistically significant.

**Table 2:** The correlation between clinicopathological parameters and HJURP expression.

Clinicopathologicalparameters		iCCA			pCCA			dCCA		
		Low	High	P	Low	High	P	Low	High	P
Age (years)	<60	10	10	0.647	16	25	0.163	4	10	0.184
	≥60	5	7		7	23		3	7	
Gender	Male	5	11	0.077	16	26	0.217	4	5	0.202
	Female	10	6		7	22		3	12	
Tumor size (cm)	<3 cm	2	4	0.468	21	35	0.076	3	14	0.058
	≥3 cm	13	13		2	13		4	3	
Histological grade	I+II	12	8	0.059	20	43	0.745	6	11	0.314
	III	3	9		3	5		1	6	
T stage	T1+2	15	10	0.006	18	29	0.140	4	10	0.941
	T3+4	0	7		5	19		3	7	
N stage	N0	11	8	0.131	11	33	0.089	7	12	0.114
	N1	4	9		12	15		0	5	
TNM stage	I+II	11	8	0.137	13	20	0.244	5	10	0.570
	III+IVA	4	9		10	28		2	7	

iCCA, intrahepatic cholangiocarcinoma; pCCA, perihilar cholangiocarcinoma; dCCA, distal cholangiocarcinoma; HJURP, holliday junction recognition protein.

### Prognostic significance of HJURP in CCA

**Figure 2:**
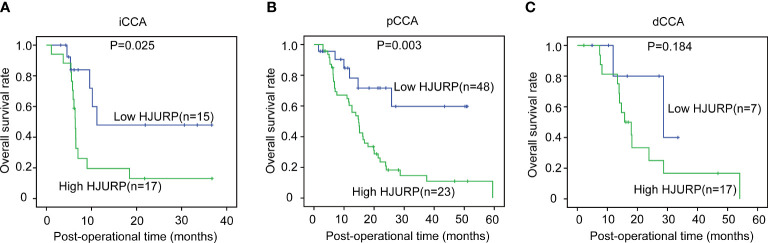
The prognostic significance of HJURP in iCCA, pCCA, and dCCA. The patients with iCCA **(A)**, pCCA **(B)**, and dCCA **(C)** were divided into low- or high-HJURP subgroups, and the statistical significance between subgroups was analyzed with the log-rank test.

The OS curves of different expression patterns of HJURP were plotted with the Kaplan–Meier method, and the prognostic significance of HJURP expression in iCCA, pCCA, and dCCA was evaluated with the log-rank test (**Table 3**). In iCCA and pCCA, patients with high HJURP had much poorer prognoses compared with others with low HJURP, suggesting that HJURP was a prognostic biomarker of iCCA and pCCA (**Figures 2A, B**). As for dCCA, the correlation between HJURP and outcome was not remarkably significant (*P* = 0.184), which may be a result of that the sample size of the dCCA cohort was not that large (n = 24)(**Figure 2C**).

**Table 3:** The prognostic significance of HJURP and clinicopathological factors of CCA.

Clinicopathological parameters		iCCA		pCCA		dCCA	
		3-year OS	P*	3-year OS	P*	3-year OS	P*
Age (years)	<60	0.251	0.919	0.285	0.667	0.205	0.372
	≥60	0.286		0.219		0.254	
Gender	Male	0.188	0.226	0.303	0.761	0.469	0.114
	Female	0.389		0.124		0	
Tumor size (cm)	<3 cm	0.667	0.063	0.311	0.014	0.222	0.395
	≥3 cm	0.122		0		0.278	
Histological grade	I+II	0.392	0.014	0.254	0.567	0.344	0.075
	III	0		0.179		0	
T stage	T1+2	0.362	0.001	0.351	0.013	0.444	0.103
	T3+4	0		0.107		0.152	
N stage	N0	0.402	0.026	0.174	0.320	0.251	0.219
	N1	0		0.380		0.400	
TNM stage	I+II	0.402	0.003	0.470	0.006	0.500	0.006
	III+IVA	0		0.088		0	
HJURP	Low	0.480	0.025	0.597	0.003	0.400	0.184
	High	0.131		0.183		0.167	

* represents analysis by the log-rank test.

iCCA, intrahepatic cholangiocarcinoma; pCCA, perihilar cholangiocarcinoma; dCCA, distal cholangiocarcinoma; HJURP, holliday junction recognition protein.

### Prognostic significance of other clinicopathological factors of CCA

**Figure 3:**
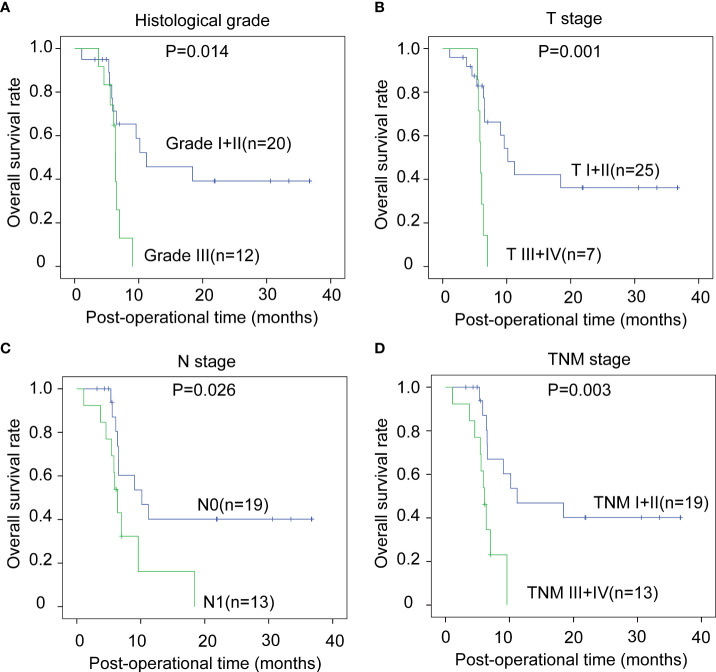
The prognostic significance of clinicopathological factors in iCCA. In iCCA, the prognosis-relevant factors included histological grade **(A)**, T stage **(B)**, N stage **(C)**, and TNM stage **(D)**. The statistical significance between subgroups was analyzed with the log-rank test.

**Figure 4:**
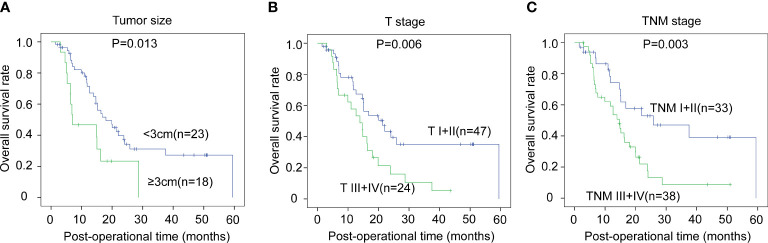
The prognostic significance of clinicopathological factors in pCCA. In pCCA, the prognosis-relevant factors included tumor size **(A)**, T stage **(B)**, and TNM stage **(C)**. The statistical significance between subgroups was analyzed with the log-rank test.

**Figure 5:**
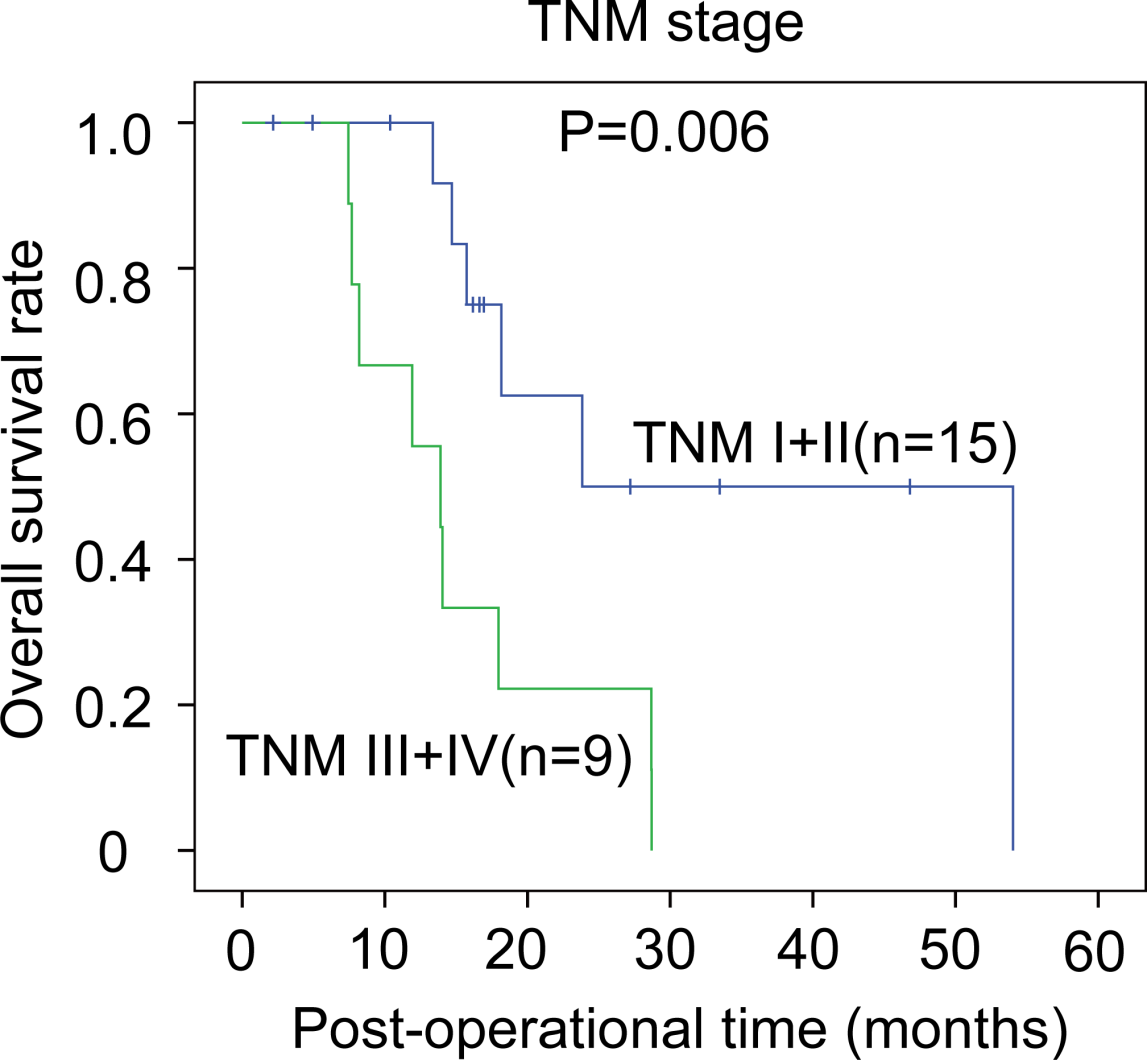
The prognostic significance of clinicopathological factors in dCCA. In dCCA, the prognosis-relevant factors included TNM stage. The statistical significance between subgroups was analyzed with the log-rank test.

The correlations between the clinicopathological factors and OS rates were also assessed with the Kaplan–Meier method. In iCCA, advanced histological grade, T stage, N stage, and TNM stage were all defined as the factors associated with poor prognosis (**Figures 3A–D**). In pCCA, the outcome-associated factors were tumor size, T stage, and TNM stage (**Figures 4A–C**). In dCCA, only TNM stage indicated the unfavorable outcome (**Figure 5**).

### HJURP is an independent prognostic biomarker of CCA

All the above clinicopathological factors were selected into the Cox-regression hazard model for multivariate analysis (**Table 4**). HJURP was identified as an independent prognostic biomarker in iCCA (*P* = 0.041, HR = 5.23, 95% CI = 1.24–22.65) and pCCA (*P* = 0.009, HR = 3.25, 95% CI = 1.33–7.91). The odds of CCA-related death for patients with high HJURP was 5.23- and 3.25-fold higher than those for patients with low HJURP. In dCCA, the independent prognostic significance of HJURP was not notable (*P* = 0.157). In addition, large tumor size (*P* = 0.012) and advanced T stage (*P* = 0.033) were also defined as independent factors suggesting poor outcome in iCCA. In pCCA and dCCA, no other clinicopathological factors were identified as independent prognostic factors in the Cox-regression hazard model.

**Table 4:** The independent prognostic factors of CCA.

Clinicopathologic parameters		iCCA			pCCA			dCCA		
		HR	95%CI	P*	HR	95%CI	P*	HR	95%CI	P*
Age (years)	<60	1			1			1		
	≥60	3.73	0.81-11.55	0.099	0.74	0.37-1.45	0.373	0.26	0.04-1.91	0.188
Gender	Male	1			1			1		
	Female	1.13	0.48-9.03	0.328	0.96	0.52-1.80	0.904	3.83	0.71-20.54	0.118
Tumor size (cm)	<3 cm	1			1			1		
	≥3 cm	17.50	1.90-161.12	0.012	1.70	0.77-3.75	0.190	0.44	0.04-5.07	0.508
Histological grade	I+II	1			1			1		
	III	0.59	0.14-2.54	0.482	1.01	0.33-3.05	0.987	6.43	0.96-42.99	0.055
T stage	T1+2	1			1			1		
	T3+4	8.67	1.19-62.99	0.033	1.63	0.81-3.28	0.171	2.75	0.79-9.57	0.112
N stage	N0	1			1			1		
	N1	1.83	0.55-6.11	0.324	0.84	0.41-1.72	0.64	1.06	0.09-12.3	0.966
HJURP	Low	1			1			1		
	High	5.23	1.24-22.65	0.041	3.25	1.33-7.91	0.009	5.91	0.51-69.05	0.157

* represents analysis by the Cox-regression model.

iCCA, intrahepatic cholangiocarcinoma; pCCA, perihilar cholangiocarcinoma; dCCA, distal cholangiocarcinoma; HR, hazard ratio; CI, confidence interval; HJURP, holliday junction recognition protein.

## Discussion

The centromere is a conserved eukaryotic protein machinery required in precision segregation of the parental genome into two daughter cells during mitosis. HJURP is a key member of the CENP-A pre-nucleosomal complex, essential for the deposition of CENP-A at the centromeres. Accordingly, HJURP should be mainly expressed in the nucleus; however, we mainly observed HJURP in cell cytoplasm. We also noticed this inconsistency in HJURP sub-localization in previous studies. In hepatocellular carcinoma, prostate cancer, ovarian cancer, and breast cancer, IHC showed that HJURP was mainly expressed in cell cytoplasm (16, 21–24); nevertheless, HJURP was mainly observed in cytoplasm in pancreatic cancer and colorectal cancer (15, 17). The underlying mechanism of this expression phenotype is still unknown. Whether HJURP has functions other than the CENP-A chaperon requires further experiments to investigate.

Emerging molecular differences between iCCA, pCCA, and dCCA were revealed, thanks to the new high-throughput techniques such as whole-genome sequencing and transcriptome sequencing. There are several molecular classifications of CCA, especially iCCA, based on the genetic alterations (25, 26). However, these molecular patterns should be verified by prognostic significance evaluation. The prognostic validation requires the cohort study of large sample size, which is difficult for CCA because of its poor radical surgical rate (27, 28). Moreover, the biological differences between pCCA and dCCA remain unclear. Interestingly, we showed that HJURP was a prognostic biomarker of iCCA and pCCA, but the prognostic significance was not observed in dCCA. Maybe the sample size of dCCA was not large enough to generate statistical difference, but this may also be a true molecular difference between pCCA and dCCA. The molecular and biological difference between pCCA and dCCA still requires a multicenter large-sample cohort study to verify.

CCA is featured with the poor prognosis and limited treatment options (29). To identify more prognostic biomarkers and promote more biomarker-driven studies is urgently needed. Till now, there is only one Food-and-Drug-Administration-approved target drug of CCA, which is pemigatinib (30). Pemigatinib is recommended to be administrated for CCA patients with FGFR2 fusion, which only accounts for 10%–15% iCCA and approximately 5% pCCA (31). Most CCA patients had no target drugs with well-accepted effect. The precision treatment of CCA requires more investigations and long-term attention. Here we identified HJURP as a prognostic biomarker of iCCA and pCCA, indicating that patients with high HJURP are high-risk and should receive more rigor post-operational examination. Our results could provide evidence for screening high-risk patients and promote the precision treatment of CCA.

In this study, we did not further investigate the molecular mechanism of HJURP-involved CCA prognosis and the role of HJURP in CCA progression. The molecular mechanism of how HJURP participated in cancer progression had various explanations. Previous studied reported that several key proteins and signaling pathways were involved in HJURP-associated cancer progression. The involved proteins include CDKN1A, SPHK1, p21, and p53, and the signaling pathways contain GSK3β/JNK signaling, MAPK/ERK1/2 signaling, AKT/GSK3β signaling pathways, and so on (16, 21, 22). The exact molecular functions and HJURP-regulated signaling in CCA need further investigations.

In summary, we detected the expression of HJURP in 127 CCA patients and demonstrated that HJURP was an independent prognostic biomarker in both iCCA and pCCA, but not in dCCA. Our results can provide more evidence of the molecular characters of different CCA subsets and suggest that patients with high HJURP are high-risk, which can guide more precision follow-up and treatment of CCA.

## Data availability statement

Publicly available datasets were analyzed in this study. This data can be found here: GSE33327,GSE132305.

## Ethics statement

This study was reviewed and approved by the study was approved with the Ethics Committee of Qilu Hospital of Shandong University and the Second Hospital Affiliated to Shandong First Medical University. The patients/participants provided their written informed consent to participate in this study.

## Author contribution

YY, JY, ZL, and WC performed the experiments, YY and PL collected the specimens and analyzed the data, and PL wrote the paper. All authors contributed to the article and approved the submitted version.

## Conflict of interest

The authors declare that the research was conducted in the absence of any commercial or financial relationships that could be construed as a potential conflict of interest.

## Publisher’s note

All claims expressed in this article are solely those of the authors and do not necessarily represent those of their affiliated organizations, or those of the publisher, the editors and the reviewers. Any product that may be evaluated in this article, or claim that may be made by its manufacturer, is not guaranteed or endorsed by the publisher.
